# Association of pre-pandemic high-density lipoprotein cholesterol with risk of COVID-19 hospitalisation and death: The UK Biobank cohort study

**DOI:** 10.1016/j.pmedr.2021.101461

**Published:** 2021-06-23

**Authors:** Camille Lassale, Mark Hamer, Álvaro Hernáez, Catharine R. Gale, G. David Batty

**Affiliations:** a Hospital del Mar Medical Research Institute, Barcelona, Spain; b CIBER of Pathophysiology of Obesity and Nutrition (CIBEROBN), Instituto de Salud Carlos III, Madrid, Spain; c Department of Epidemiology and Public Health, University College London, UK; d Division of Surgery and Interventional Sciences, University College London, London, UK; e Centre for Fertility and Health (CeFH), Norwegian Institute of Public Health, Oslo, Norway; f Blanquerna School of Health Sciences, Universitat Ramon Llull, Barcelona, Spain; g August Pi i Sunyer Biomedical Research Institute, Barcelona, Spain; h MRC Lifecourse Epidemiology Unit, University of Southampton, UK; i Lothian Birth Cohorts, Department of Psychology, University of Edinburgh, UK

**Keywords:** HDL-C, COVID-19, Cohort study, UK Biobank

## Abstract

There is growing evidence of, and biological plausibility for, elevated levels of high-density lipoprotein cholesterol (HDL-C) being related to lower rates of respiratory disease. We tested whether pre-pandemic HDL-C within the normal range is associated with subsequent COVID-19 hospitalisations and death. We analysed data on participants from UK Biobank, a prospective cohort study, baseline data for which were collected between 2006 and 2010. Follow-up for COVID-19 was via hospitalisation records (1845 events in 317,306 individuals) and a national mortality registry (458 deaths in 317,833 individuals). After controlling for a series of confounding factors which included health behaviours, inflammatory markers, and socio-economic status, higher levels of HDL-C were related to a lower risk of later hospitalisation. The effect was linear (p-value for trend 0.001), whereby a 0.2 mmol/L increase in HDL-C was associated with a 7% lower risk (odds ratio; 95% confidence interval: 0.93; 0.90, 0.96). Corresponding relationships for mortality were markedly weaker, such that statistical significance at conventional levels were not apparent for both the linear trend (p-value 0.25) and the odds ratio per 0.2 mmol/L increase (0.98; 0.91, 1.05). While our finding for HDL-C and hospitalisations for COVID-19 raise the possibility that favourable modification of this cholesterol fraction via lifestyle changes or drug intervention may impact upon the risk of the disease, it warrants testing in other studies.

## 1 Introduction

High-density lipoprotein cholesterol (HDL-C) has traditionally been linked with coronary heart disease and stroke (Gordon et al., 1989). While conventional epidemiological studies consistently demonstrate that elevated levels of this cholesterol fraction confer protection against vascular events (Di Angelantonio et al., 2009), support for such a gradient has been lacking in people genetically predisposed to low concentrations of HDL-C (Frikke-Schmidt et al., 2008), Mendelian randomisation studies (Holmes et al., 2015), and randomized clinical trials utilising HDL-C–elevating medication (Keene et al., 2014).

More recently, HDL-C has been implicated in the pathogenesis of other health endpoints, including infectious disease. While most investigators testing the link between HDL-C and infection have done so in prognostic studies of patients groups (Shor et al., 2008), in one of the few cohort analyses of apparently healthy individuals, people with lower levels of baseline HDL-C experienced a greater subsequent risk of hospitalisation for gastroenteritis, urinary tract infection, and bacterial pneumonia (Madsen et al., 2018). Plausible mechanisms include the HDL-C–mediated sequestration of pathogen-associated lipids, and regulation of immune cells proliferation, maturation, and function which results in neutralization or clearance of pro-inflammatory endotoxins (Catapano et al., 2014).

This evidence base raises the possibility of a link between HDL-C and COVID-19, the disease caused by severe acute respiratory syndrome coronavirus 2. Using UK Biobank, a prospective cohort study, we have recently shown that an unfavourable pre-pandemic vascular risk factor profile – low HDL-C included – is associated with a higher risk of hospitalization for COVID-19 (Batty and Hamer, 2020). Whether HDL-C across the normal range offers predictive capacity for COVID-19 hospitalisations is, however, untested, and this is the purpose of the present study. Further, as the present pandemic has unfolded, this cohort has accumulated sufficient deaths from this disease to facilitate analyses with the aim of corroborating any associations with hospitalisations.

## 2 Methods

### 2.1 Study population

We used data from the UK Biobank, a prospective cohort study (Sudlow et al., 2015), baseline data collection for which took place between 2006 and 2010 across centres in the UK, yielding a sample of 502,655 people (448,919 from England) aged 40–69 years. Ethical approval was provided by the North-West Multi-centre Research Ethics Committee (11/NW/0382; 16/NW/0274).

### 2.2 Baseline data collection

At baseline, non-fasting venous blood samples were drawn and assayed for total cholesterol, HDL-C, and triglycerides using a Beckman Coulter AU5800 analytical platform. Low density lipoprotein (LDL)-cholesterol values were calculated using the Friedewald equation (Friedewald et al., 1972). Total blood count (leukocyte, platelet, haemoglobin) as markers of immune function were analysed using an automated Coulter LH 750. Physician-diagnosed cardiovascular disease (heart attack, angina, stroke), diabetes, cholesterol-lowering drugs use, cigarette smoking, alcohol intake, highest educational attainment, ethnicity (Lassale et al., 2020), number of people living in the household, and physical activity in the prior month were self-reported using standard enquiries (Batty et al., 2020a). Body mass index was computed using direct measurements of height and weight using the usual formulae (Hamer et al., 2020b). Hypertension was defined as elevated measured blood pressure (≥140/90 mmHg) and/or use of anti-hypertensive medication. The Townsend index of neighbourhood deprivation was based on postcode linkage (Batty et al., 2020a). Provided by Public Health England, data on COVID-19 status in hospitalised patients in England covered the period 16th March until 24th October 2020. Tests were performed in accredited laboratories on samples from combined nose/throat swabs using real time polymerase chain reaction. Participants were also linked to long-standing national mortality records from which death from COVID-19 for the period 1st March 2020 to 24th January 2021, was denoted by the emergency International Classification of Disease (version 10) code U07.1 (COVID-19, virus identified).

### 2.3 Statistical analyses

We used logistic regression analyses to compute odds ratios with accompanying 95% confidence intervals to summarise the relation between HDL-C and later COVID-19 hospitalisation or death. In doing so, we created an HDL-C variable with eight categories which was designed to examine the shape of the HDL-C–COVID-19 relationship (<1.0 [referent], 1.0 to < 1.2, 1.2 to < 1.4, 1.4 to < 1.6, 1.6 to < 1.8, 1.8 to < 2.0, 2.0 to < 2.2, ≥2.2 mmol/L); in the analyses of COVID-19 deaths, the latter two categories were collapsed owing to a lower number of events. With preliminary analyses suggesting a linear gradient, we were then able to summarise the relationship for a unit change in HDL-C (0.2 mmol/L increase). We first adjusted for age and sex (comparator model) and then, in the multivariable model, a series of covariates which included inflammatory markers, lifestyle factors, and socioeconomic circumstances.

## 3 Results

### 3.1 HDL-C and hospitalisation for COVID-19

**Fig. 1:**
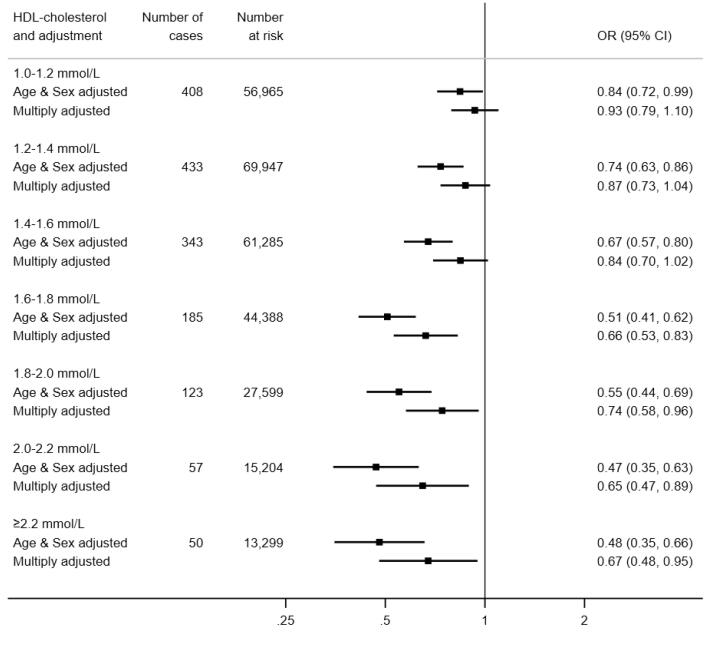
HDL-C concentration (2006–10) and risk of subsequent hospitalisation for COVID-19 (2020) in UK Biobank (N = 317,306). Multiply adjusted odds ratios are adjusted for age, sex, ethnicity, education, number in household, area deprivation, body mass index, leisure time physical activity, alcohol intake, smoking habit, diagnosed diabetes, cardiovascular disease, or hypertension, cholesterol-lowering medication, LDL-cholesterol, triglycerides, haemoglobin, white blood cell, and platelet count. The reference group is HDL-C <1.0 mmol/L, comprising 28,619 participants at risk of whom 246 were hospitalised.

In 317,306 (171,466 women) participants with complete data on baseline covariates, there were 1845 hospitalisations for COVID-19 (899 in women) during the surveillance period. As illustrated in Fig. 1, in age- and sex-adjusted analyses, relative to the group with the lowest concentration of HDL-C, those in the highest experienced around half the risk of hospitalization for COVID-19 (odds ratio; 95% confidence interval: 0.48; 0.35, 0.66). There was also evidence of a stepwise relationship (p-value linear trend < 0.001) such that lower disease risk was apparent in people with higher level of this cholesterol fraction. Summarising this trend, a 0.2 mmol/L increase in HDL-C was associated with a 12% lower risk of subsequent hospitalisation (0.88; 0.86, 0.91). Adjusting for an array of confounding factors – health behaviours, inflammatory markers, and socio-economic status – resulted in a shallower HDL-C–COVID-19 gradient but the linear association remained (p for linear trend 0.001) with a 0.2 mmol/L increase in the cholesterol fraction now corresponding to a 7% lower risk of the disease (0.93; 0.90, 0.96).

### 3.2 HDL-C and death from COVID-19

**Fig. 2:**
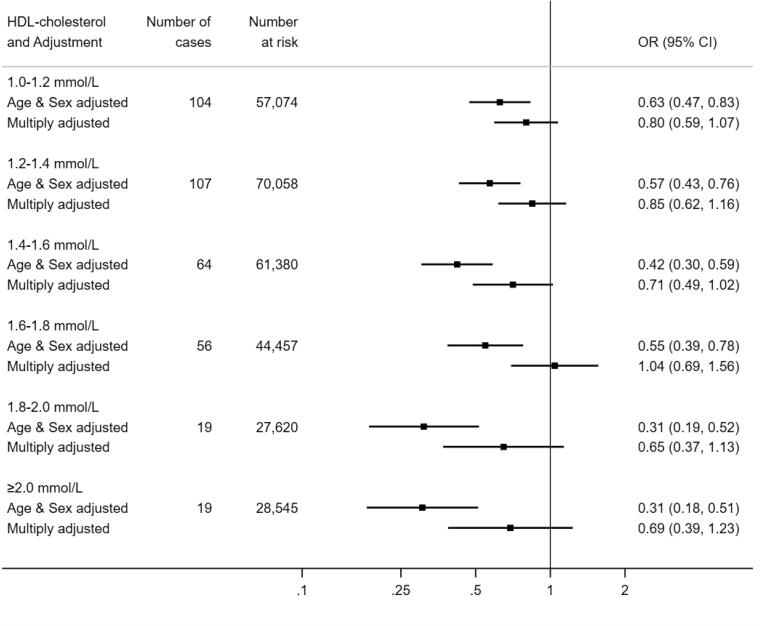
HDL-C concentration (2006–10) and risk of death from COVID-19 (2020) in UK Biobank (N = 317,827). Multiply adjusted odds ratios are adjusted for age, sex, ethnicity, education number in household, area deprivation, body mass index, leisure time physical activity, alcohol intake, smoking habit, diagnosed diabetes, cardiovascular disease, or hypertension, cholesterol-lowering medication, LDL-cholesterol, triglycerides, haemoglobin, white blood cell, and platelet count.  The reference group is HDL-C <1.0 mmol/L, comprising 28,699 participants at risk of whom 89 died of COVID-19.

Next, we tested the results for HDL-C and COVID-19 mortality in 317,833 participants (171,677 women) in whom there were 458 COVID-19 deaths (143 in women) during the surveillance period. As apparent in Fig. 2, the shape of the HDL-C relation with mortality was similar to that seen in the prior analyses for hospitalisations. Thus, after controlling for age and sex, relative to the group with the lowest concentration of HDL-C, those individuals in the group with the highest HDL-C concentration (≥2.0 mmol/L) had a third of the risk of death ascribed to COVID-19 (odds ratio; 95% confidence interval: 0.31; 0.18, 0.51). Again, there was evidence of a dose–response effect across the full range of HDL-C values (p-value for trend < 0.0001) whereby an increase in HDL-C of 0.2 mmol/L produced a 15% lower risk of mortality (0.85; 0.80, 0.90). As apparent from the breadth of the confidence intervals for several point estimates, however, some of these analyses had lower precision owing to the lower number of deaths. Multiple adjustment for covariates led to marked attenuation of the magnitude of the HDL-C–death relation such that statistical significance at conventional levels was lost for both the linear trend (p-value 0.25) and the odds ratio per 0.2 mmol/L increase (0.98; 0.91, 1.05).

### 3.3 Discussion

To the best of our knowledge, this is the first study to examine the shape of the relationship between pre-pandemic HDL-C levels and risk of later COVID-19 events. Our salient finding was that, net of an array of confounding factors, higher concentrations of HDL-C were associated with protection against hospitalisation for the disease but not with death from it. We are unclear why there was little evidence of a relation with death, the more severe proxy for COVID-19. Giving us some confidence in these results, in earlier follow-up, we have reported what are now regarded as established associations of classic vascular risk factors such as raised levels of glycosylated haemoglobin (Hamer et al., 2020a), body weight (Hamer et al., 2020b), and blood pressure (Batty and Hamer, 2020) with COVID-19 hospitalisations, as apparent in studies based in the US (Richardson et al., 2020), Italy (Grasselli et al., 2020), China (Wu et al., 2020, Zhou et al., 2020), and Brazil (Baqui et al., 2020). We have also shown higher rates of hospitalisation for the disease with increasing age, male sex, socioeconomic disadvantage (Batty et al., 2020a), and ethnic minority groups (Lassale et al., 2020).

### 3.4 Comparison with existing studies

Levels of HDL-C appear to change in the presence of COVID-19, such that, relative to healthy controls, patients with the disease have lower levels of HDL-C concentrations in conjunction with acute elevation in systemic inflammatory markers (Sorokin et al., 2020, Wei et al., 2020). While a lowering of HDL-C levels appears to be a consequence of COVID-19, the reverse, whereby pre-pandemic HDL-C levels may offer some predictive capacity for this condition, has been little examined. Taking a Mendelian Randomisation approach, investigators using UK Biobank data have shown an inverse association between baseline HDL-C and hospitalisations for any infectious disease up to 10 years later (Trinder et al., 2020). In contrast, using a conventional epidemiological study design, analyses based on two Danish community-based, prospective cohort studies with up to 20 years of follow-up revealed a ‘U’-shaped relationship whereby the greatest risk of hospitalisation for any infection was apparent at opposing ends of the HDL-C continuum (Madsen et al., 2018). There was no clear evidence of such a quadratic effect in the present study.

#### 3.4.1 Mechanisms of effect

Potential mechanisms for the inverse HDL-C and COVID-19 hospitalisation association include the anti-inflammatory properties of this lipoprotein which may have relevance for COVID-19 complications. Further, HDL-C can act as a direct modulator of immunity by inhibiting haematopoietic stem cells proliferation and affecting the activity and function of immune cells by changing the cholesterol content of the lipid rafts in immune cell membranes (Catapano et al., 2014). A direct binding and neutralization of viral particles by HDL-C may also underpin the observed association (Kane et al., 1979).

#### 3.4.2 Study strengths and weaknesses

The strengths of this study include the measurement of biomarkers that preceded the onset of COVID-19, so ruling out reverse causality. While large, the study sample is also well-characterised. That UK Biobank participants represent only 6% of the target population, however, means that the present data cannot be used to estimate prevalence or incidence in the general population, although established risk factor associations appear generalizable (Batty et al., 2020b). HDL-C levels were measured up to 14 years before COVID-19 case assessment and this raises concerns about their utility for current levels; however, in a reassessment a mean of 4.4 years after baseline examination they showed high test–retest stability (correlation coefficient 0.85, p < 0.001) in a subsample (N = 13,430). While the HDL-C–COVID-19 gradient was robust to the adjustment of various covariates, it is plausible that unmeasured confounding factors might explain the association. To this extent, because the data are observational, we cannot be dogmatic about causality. Further scrutiny of our results, including the application of the Mendelian Randomisation approach where a genetic proxy for HDL-C is used as the exposure of interest, is required.

In conclusion, our findings raise the possibility that favourable modification of this cholesterol fraction via lifestyle changes or drug intervention may impact upon the risk of hospitalisation for COVID-19 . These observations warrant testing using other study designs.

## Funding

CL is supported by the Beatriu de Pinós postdoctoral programme of the Government of Catalonia's Secretariat for Universities and Research of the Ministry of Economy and Knowledge (2017-BP-00021); GDB by the Medical Research Council (MR/P023444/1) and the US National Institute on Aging (1R56AG052519-01; 1R01AG052519-01A1); and MH through a joint award from the Economic Social Research Council and Medical Research Council (RES-579-47-0001)

## Declaration of Competing Interest

The authors declare that they have no known competing financial interests or personal relationships that could have appeared to influence the work reported in this paper.
